# Dielectric and relaxation properties of composites of epoxy resin and hyperbranched-polyester-treated nanosilica

**DOI:** 10.1039/c8ra05846f

**Published:** 2018-08-31

**Authors:** Guoqing Yang, Junda Cui, Yoshimichi Ohki, Deyi Wang, Yang Li, Kai Tao

**Affiliations:** State Key Laboratory of Eco-hydraulics in Northwest Arid Region of China, Xi'an University of Technology Xi'an 710048 P. R. China yanggq@xaut.edu.cn; Department of Electrical Engineering and Bioscience, Waseda University Tokyo 169-8555 Japan w.iac17125@kurenai.waseda.jp; Research Institute for Material Science and Technology, Waseda University Tokyo 169-0051 Japan yohki@waseda.jp

## Abstract

Hyperbranched polyester is effective for enhancing molecular bond strength and improving the mechanical behavior of nanofilled polymers. This study examines the dielectric and polarization relaxation characteristics of epoxy resin composites filled with nanosilica 30 nm in diameter, which is treated by terminal carboxyl hyperbranched polyester. TEM and SEM analysis indicate that the nanosilica surface is grafted with a functional polymer layer ranging in thickness from several to tens of nanometers, and the nanosilica agglomeration in epoxy resin is remarkably inhibited. Measurements of thermally stimulated depolarization current and differential scanning calorimetry show that, deep traps with an energy of 1.09 eV are present in the nanocomposites, and the glass transition temperature (*T*_g_) is increased by 11 °C at most at filler concentrations from 1 to 7 wt%. Moreover, the room-temperature relative permittivity and dielectric loss factor of the composites at 50 Hz are decreased by 0.22 and 1.3‰, respectively. Conductivity at 10 mHz to 1 kHz and dc conductivity are also significantly decreased when the operating temperature is below *T*_g_. The polarization relaxation process of the nanocomposite is dominated by regional carrier migration, interfacial and dipole polarization. The relaxation frequency of dipole polarization at high temperature (>*T*_g_) is transformed to satisfy the Vogel–Tammann–Fulcher law. This research suggests that both the dielectric and the polarization relaxation properties of the epoxy resin composites can be modified by filling hyperbranched-polyester-treated nanosilica, because it enhances the bond strength of the inorganic–organic interface and enlarges the molecular scale of the composites *via* cross-linking reactions.

## Abstract 2

Dielectric and relaxation properties of a nanofilled epoxy composite can be effectively modified by nanofiller treatment with a terminal carboxyl hyperbranched polyester.

## Graphical abstract

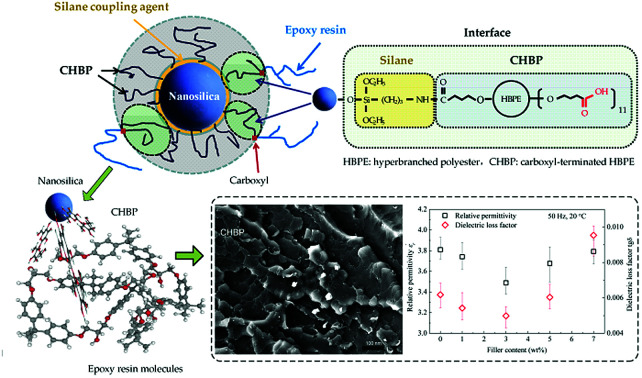

## 1. Introduction

Nanosized inorganic fillers are often used to modify the insulating,^1^ mechanical,^2^ thermal,^3,4^ and flame-retardant^5^ abilities – among others – of polymer composites.^6–8^ Owing to their high surface energy and hydrophobicity, nanofillers require pretreatment before being mixed with epoxy resin. Among many surface-modification methods for the nanofiller, surface grafting is one of the most popular chemical methods. Silane or other similar coupling agents are widely used to treat inorganic nanofillers because they contain both hydrophilic and hydrophobic groups, located at opposite terminals. They can act as bridges and combine inorganic particles with organic polymer molecules. However, owing to the linear structures and low molecular weights of these conventional coupling agents, the interaction or modification area is not wide, and the bond strength is inadequate, which limits the ability to improve the behavior of the composites. Regarding this issue, considerable research has been conducted to find more effective surface modifiers and modification methods for inorganic nanofillers.^9,10^

Hyperbranched polyester is a kind of highly branched three-dimensional polyester possessing numerous dendritic structures and active terminal radicals. It has been used to modify inorganic fillers of polymer composites in recent years. In particular, the improvement of the mechanical and thermal properties has been most actively investigated.^3,11,12^ In our previous study, the surface modification treated by carboxyl-terminated hyperbranched polyester (CHBP) significantly inhibited the agglomeration of nanosilica in epoxy resin and enhanced the tension and alternating-current (ac) breakdown strength of the composite.^13^ Owing to its dendritic molecular structure and active terminal groups, the hyperbranched polyester endows nanofillers with a higher probability of interacting with the epoxy matrix and a stronger bond force. However, little is known about the influence of hyperbranched polyester surface modification on the dielectric properties of nanofilled polymers. It is believed that the trap distribution and charge mobility of nanofilled composites are remarkably influenced by the interaction zone around fillers.^14–17^ In addition to the enhancement of the toughness of epoxy resin, the higher bond energy between nanofillers and epoxy molecules should exert various influences on the dielectric properties and molecular motions.^18^ The purpose of this study is to investigate the dielectric and relaxation properties of epoxy composites filled with nanosilica, focusing on the effects of surface treatment with the hyperbranched polyester.

## 2. Experimental

**Fig. 1:**
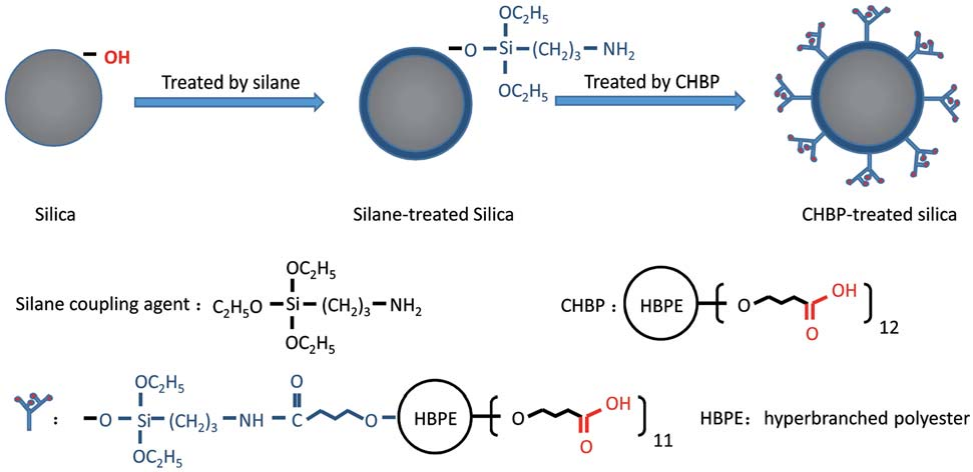
Sequential reactions in the process of surface modification of nanosilica.

We purchased nanosilica, of a spherical shape with a nominal diameter of 30 nm, from Aluminum Corporation of China. The nanosilica was treated in two steps. As shown in Fig. 1, nanosilica particles were first treated with a silane coupling agent (aminopropyltriethoxy silane). Next, the silane-treated nanosilica was treated with CHBP (Hyper C102, Wuhan Hyperpolymer), which has a molecular weight of 2600 and 12 terminal carboxyl groups.

Nanosilica particles were mixed with ethanol and aminopropyltriethoxysilane (3-APTES). The weights of ethanol and 3-APTES are double and 10% of silica's. After mixing, the hybrid solution was stirred for 40 min at 20 °C, and then heated to 100 °C for 20 min to remove the solvent. Next, the silane-treated silica was gradually added into the mixture of 0.5 wt% of CHBP, 300 wt% of acetone and 0.1 wt% of *p*-toluenesulfonic acid (PTSA). Here, the weight of the dried silica particles is regarded as 100%. After reacting with the solution for 40 min, silica was dried again under 100 °C.

During the first step of surface treatment, silane chains with a molecular weight of approximately 260 react with surface hydroxyl radicals on the surfaces of the nanosilica and are grafted on the surfaces. Owing to the existence of amino groups in the grafted silane molecule, CHBP can be successfully grafted onto the nanosilica surfaces *via* the reaction between amino and carboxyl groups. After two steps of surface modification, the surface activity of nanosilica particles is modified significantly by the presence of the dendritic structures and terminal carboxyl acids.

As a next step, epoxy resin nanocomposites were fabricated using the silica nanoparticles modified as previously mentioned. Bisphenol-A epoxy resin (E51, Sinopec) was used as base matrix resin. Methyl tetrahydrophthalic anhydride and 2,4,6-tris(dimethylaminomethyl)-phenol were used as a hardener and a curing accelerator, respectively. The nanosilica content was set to 0, 1, 3, 5, or 7 weight percent (wt%) of epoxy resin. After complete curing, the epoxy resin composites were shaped into circular sheets 0.5 mm thick and 30 mm in diameter.

Before electric testing of epoxy composite sample, its surface composition was scanned by XPS (AXIS-ULTRA, Kratos). Distribution of nanofillers in epoxy composite was also observed by SEM (JSM-6700F, JEOL).

For insulating polymers, measurements of thermally stimulated depolarization current (TSDC) often provides useful information about structural, thermal, and electrical properties.^19,20^ Therefore, the composite samples were polarized at a direct-current (dc) electric field of 20 kV mm^−1^ at 100 °C and then heated again from 10 to 200 °C to measure their TSDC. Using a digital electrometer (R8252, Advantest), the dc conductivity of the composite was measured at an applied electric field of 10 kV mm^−1^ and 20 °C. The complex permittivity and glass transition temperature (*T*_g_) of the composite samples were observed using a dielectric spectrometer (SI1260, Solartron) and a differential scanning calorimeter (DSC, TA-60WS, Shimadzu), respectively. For the DSC test, the temperature was increased at a rate of 10 °C min^−1^.

## 3. Results

**Fig. 2:**
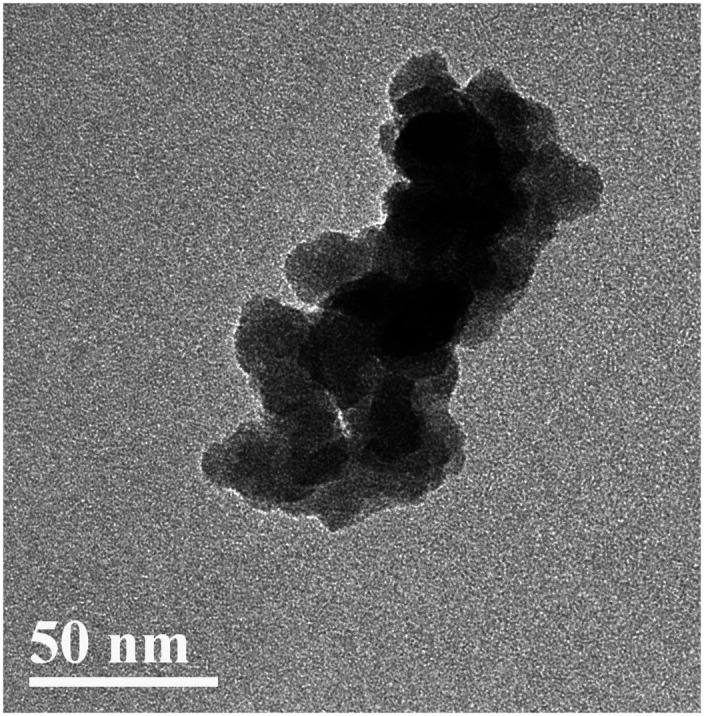
TEM image of functional nanosilica.

**Fig. 3:**
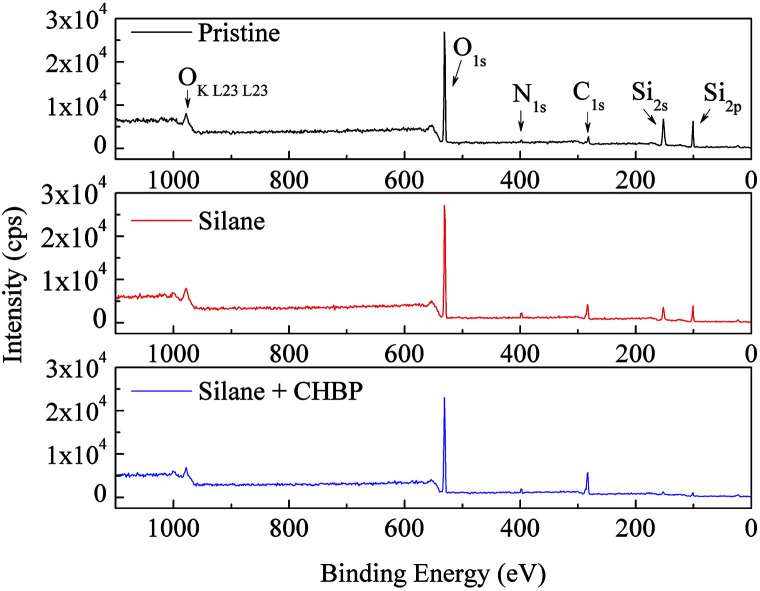
XPS spectra of nanofillers.

After two steps of grafting reactions, nanosilica surface would be covered by silane and CHBP layers. Fig. 2 shows the TEM image of treated nanosilica. It is clear that core–shell structured functional nanosilica was fabricated *via* grafted silane and CHPB molecules, and the thickness of CHBP functional layer ranges from several to tens of nanometers. Fig. 3 shows the XPS spectra of nanofiller surfaces. It is indicated that the surface composition of nanosilica, including Si, O, C and N concentration, has been greatly changed by silane and CHBP grafting reactions.

**Fig. 4:**
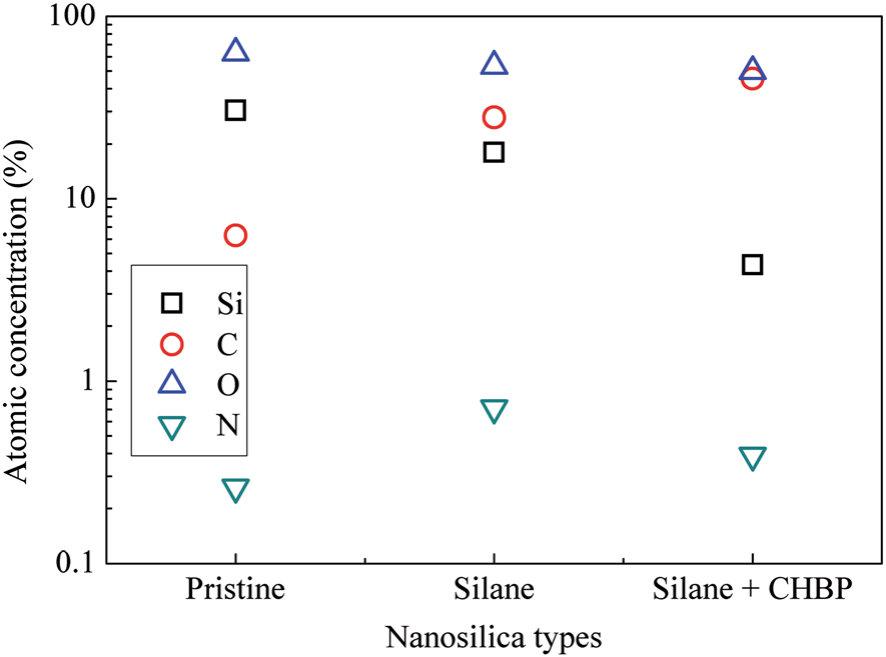
Atomic concentration of main elements on nanosilica surface.

Fig. 4 shows the calculated atomic concentration of nanosilica surface based on XPS spectrum. It is obvious that Si concentration on nanofiller surface was decreased from 30.54% to 18.01% and 4.33% by surface modification of silane and CHBP solution. Besides this, C ratio was gradually increased from 6.28% to 27.91% and 45.45%. N concentration was firstly increased to 0.71% by silane modification and then was decreased to 0.39% after further CHBP treatment. Because there are no Si and N elements in CHBP molecules, and N only exists in silane, the decrease in concentration of Si and N atoms on nanosilica surface means that nanosilica was nearly completely enveloped by CHBP layer. In addition, owing to the fact that the thickness of grafting CHBP layer is less than the effective scanning depth of XPS test, around 5 nm, very few of Si and N existed in nanosilica surface or silane grafting layer were still found in XPS spectrum.

**Fig. 5:**
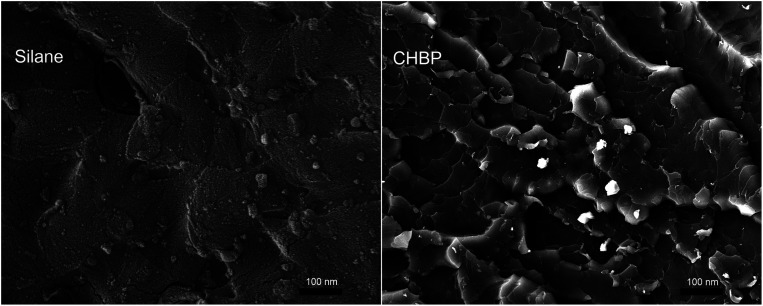
SEM pictures of cross section of epoxy composite samples.

Fig. 5 shows the cross section view of epoxy composite filled with 3 wt% of silane-treated and CHBP-treated nanosilica. It is clear that nanofillers distribute in epoxy composite without obvious agglomeration. It also can be found that for epoxy composite filled with silane-treated nanosilica, its fracture mode shows as brittle fracture, as shown in the left picture of Fig. 5. However, owing to the enhancement of CHBP-treated nanofiller, the failure mode of epoxy composite is changed from brittle to typical ductile fracture.

**Fig. 6:**
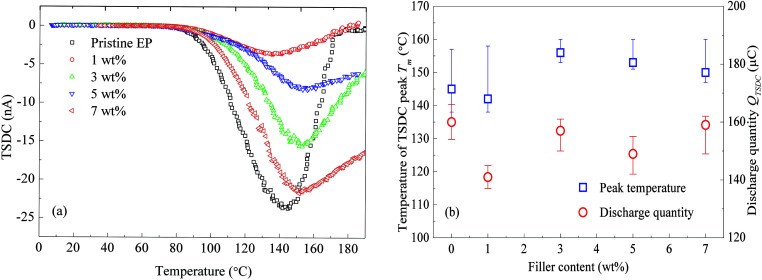
TSDC measured in epoxy resin filled with nanosilica: (a) the temperature dependence of the TSDC; (b) the peak temperature and integrated discharge quantity as a function of the nanosilica content.

Epoxy resin composites filled with 0, 1, 3, 5, and 7 wt% of nanosilica were prepared for the TSDC test. The experimental results are shown in Fig. 6. Regardless of the filler content, the composites show a peak around 140 to 160 °C. The filler-content dependence of the peak temperature is shown in Fig. 6(b). The spectra that do not decrease to zero at 200 °C were extrapolated by fitting Gaussian curves.

Fig. 6(b) indicates that with the increase of the filler content, the peak temperature of the TSDC monotonically increases from 138 to 161 °C. Although the filler-content dependence of the charge quantity released as TSDC (*Q*_TSDC_) is not simple, the values of *Q*_TSDC_ emitted from the composite filled with nanosilica are always smaller than the value observed for the pristine epoxy resin. These results suggest that the TSDC is significantly suppressed by the addition of CHBP-treated nanosilica.

**Fig. 7:**
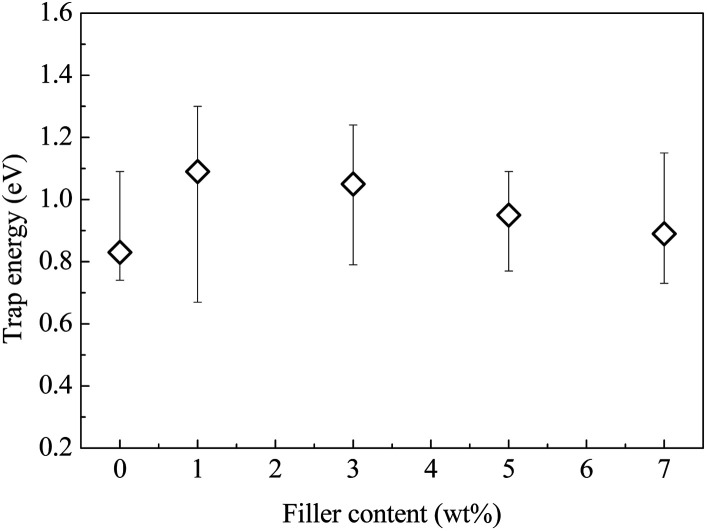
Trap energy as a function of the filler content.

It is known that the TSDC in an insulating polymer arises mainly as a result of the thermally assisted detrapping of injected charges or the thermal relaxation of the dipolar orientation.^21^ Using the initial rise method,^22^ the activation energy of the thermal process can be calculated. Fig. 7 shows the calculated results based on the TSDC curves shown in Fig. 6(a).

The results shown in Fig. 7 indicate that the trap energy of the composite is obviously modified by the addition of CHBP-treated nanosilica. For the pristine epoxy resin, the activation energy is around 0.83 eV. At a filler content of approximately 1 to 7 wt%, it ranges from 0.89 to 1.09 eV. This means that the depolarization of dipoles and the release of trapped charges become more difficult in the nanofilled epoxy resin. While the trap energy is maximized at the filler content of 1 wt%, it gradually decreases with the further increase of the filler content and reaches a similar value to the pristine sample at 7 wt%.

**Fig. 8:**
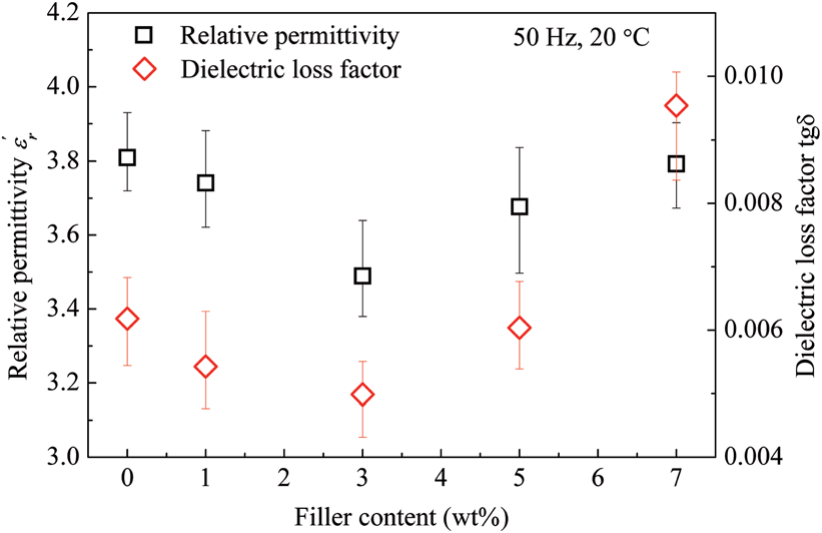
Relative permittivity and dielectric loss factor at 50 Hz and 20 °C.

Fig. 8 gives the real part of the complex relative permittivity 
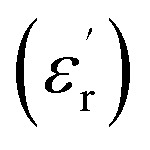
 and dielectric loss factor (tg *δ*) of nanocomposites at 50 Hz and 20 °C. It shows that with the increase in filler content, the permittivity constant and dielectric loss both decrease obviously. At 3 wt%, the nanocomposites own the lowest permittivity and dielectric loss. At 7 wt% of filler content, the value of 
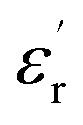
 and tg *δ* approaches and exceeds that of pristine epoxy resin, respectively.

**Fig. 9:**
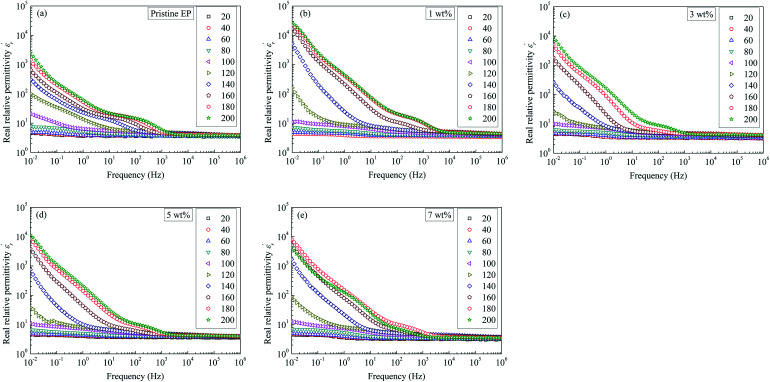
Real dielectric permittivity as a function of frequency observed for: (a) pristine epoxy resin; (b) 1 wt%; (c) 3 wt%; (d) 5 wt%; (e) 7 wt%.

Fig. 9 shows relative permittivity of epoxy resin composites measured at different temperatures and frequencies. There is a sudden increase in 
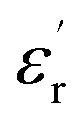
 between 100 and 1 kHz. While 
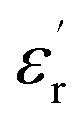
 decreases with the increase in frequency (*f*), the loading of CHBP-treated nanosilica seems to suppress the appearance of a very high 
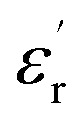
 at a low frequency and below 120 °C. However, at a high temperature, the 
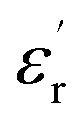
 of the composites filled with nanosilica is far larger than that of the pristine epoxy resin.

**Fig. 10:**
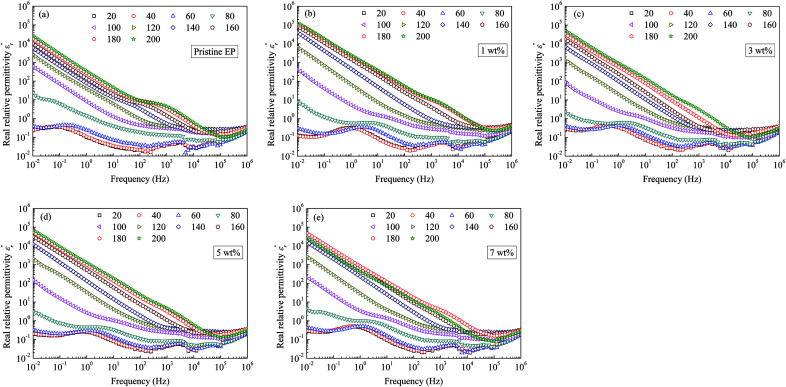
Imaginary dielectric-permittivity values as a function of frequency observed for: (a) pristine epoxy resin; (b) 1 wt%; (c) 3 wt%; (d) 5 wt%; (e) 7 wt%.

Fig. 10 shows the imaginary part of the relative permittivity 
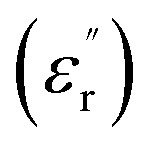
 measured for pristine and nanofilled samples. As discussed in the next section, the imaginary value of the relative permittivity mainly reflects the transport properties of charge carriers, especially in insulating polymers at a low frequency and a high temperature.^23^Fig. 10 indicates that a slope-like curve corresponding to the conduction process gradually emerges if the composite samples are heated. The 
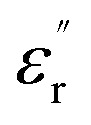
 of the nanofiller epoxy resin at a low frequency is smaller than that for pristine epoxy resin at a temperature below 120 °C.

**Fig. 11:**
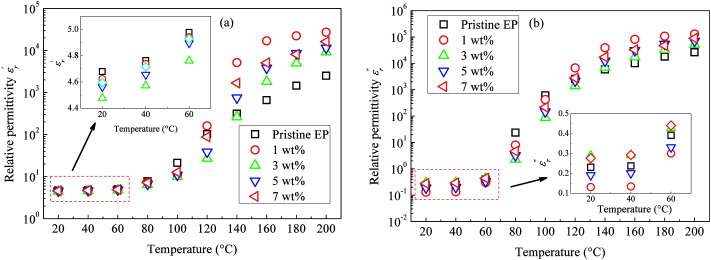
Relative permittivity as a function of temperature: (a) real part; (b) imaginary part.

Fig. 11 shows the temperature dependence of the 
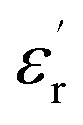
 and 
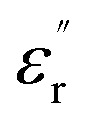
 of the composite samples at 0.01 Hz. Clearly, the composites filled with CHBP-treated nanosilica exhibit a reduced relative permittivity and dielectric loss at a low temperature. This phenomenon becomes more obvious for the 3 and 5 wt% samples. The value of 
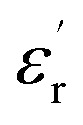
 of the nanofilled composites is approximately 0.2 smaller than that of the unfilled epoxy resin. However, at a high temperature, values of 
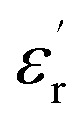
 and 
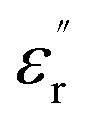
 of the pristine epoxy resin are remarkably smaller than those of all the nanofilled composites.

For obtaining the transition frequency of the dielectric permittivity at different temperatures, the use of the electric modulus, which is the inverse of the complex relative permittivity, is more effective than the use of the original permittivity. In the case where 
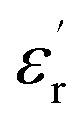
 and 
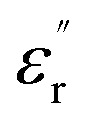
 satisfy the Cole–Cole relation, the electric modulus (M*) can be expressed by eqn (1):^24^1
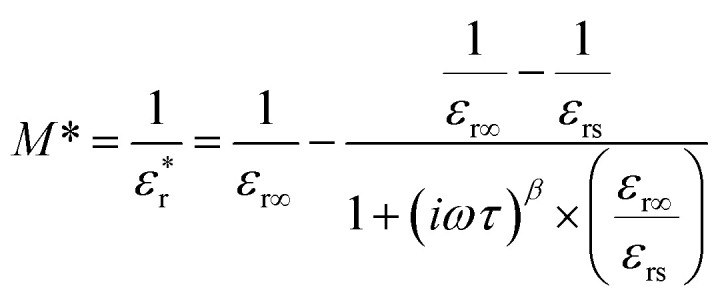
where 
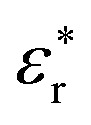
 is the complex relative permittivity, *ε*_r∞_ and *ε*_rs_ are the values of the relative permittivity at infinitely high and near-zero frequencies, respectively, *τ* is the relaxation time, *β* is the shape parameter, and *ω* is the angular frequency.

**Fig. 12:**
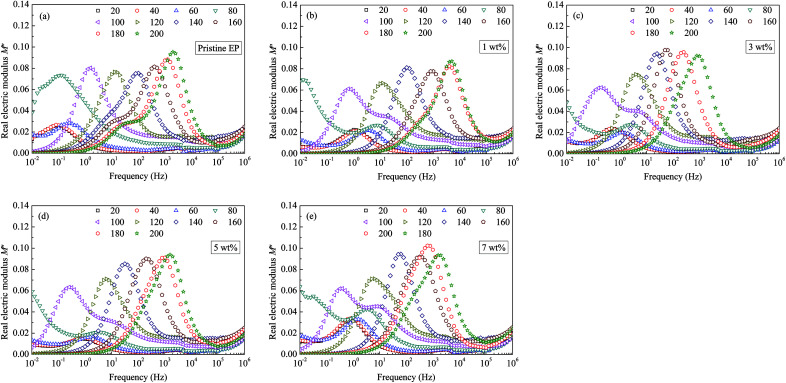
Imaginary modulus values as a function of frequency observed for: (a) pristine epoxy resin; (b) 1 wt%; (c) 3 wt%; (d) 5 wt%; (e) 7 wt%.

Considering that the real part does not exhibit clear features, which is similar to our previous studies, only the imaginary part of the electric modulus is shown in Fig. 12. Clear loss peaks appear in the spectra shown in Fig. 12. If the sample temperature is <100 °C, two independent peaks appear in the curve of the imaginary electric modulus (*M*′′), and the loss peak is wider than the one at a higher temperature. Moreover, with the addition of CHBP-treated nanosilica, the relaxation peak frequency decreases, so that the *M*′′ spectra appear to move towards the low-frequency direction. Moreover, if the filler content is more than 5 wt%, the decrease of relaxation peak frequency is not obvious any more.

**Fig. 13:**
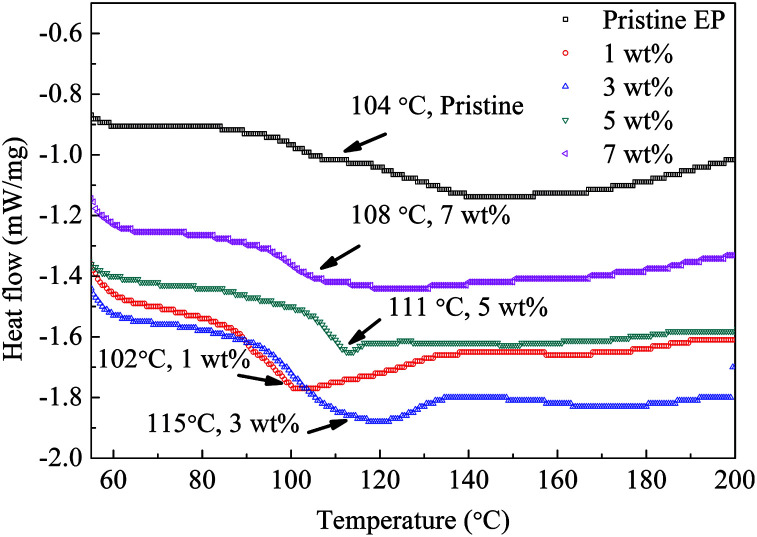
DSC results for epoxy resin composites.

Fig. 13 shows the DSC test results of the composite samples. The value of *T*_g_ of the epoxy resin composite is obviously improved by filling nanosilica by 3, 5 or 7 wt% of. For the 1 wt% sample, *T*_g_ is similar to that of pristine epoxy resin. The heat-flow curve of the pristine epoxy resin clearly indicates two separated glass transition processes and a wide transition-temperature range. However, for the nanofilled samples, only one drop of the heat flow is observed, and the transition-temperature width is reduced.

**Fig. 14:**
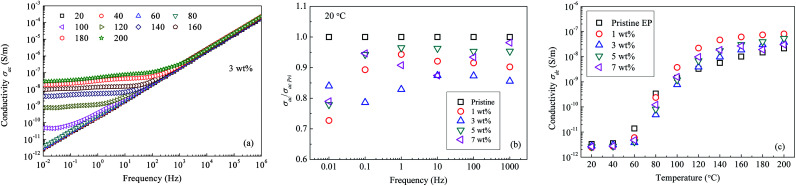
Conductivity of epoxy resin composites: (a) *σ*_ac_ as a function of frequency; (b) nanocomposite *σ*_ac_*vs.* pristine *σ*_ac_; (c) *σ*_dc_ as a function of temperature.

Fig. 14(a) shows the ac conductivity (*σ*_ac_) values for the composite filled with 3 wt% of nanosilica. The conductivity *σ*_ac_ increases with an increase in frequency of the applied electric field. Additionally, if the temperature is <100 °C, the logarithmic value of *σ*_ac_ is proportional to the field frequency. If the temperature of the composite exceeds 100 °C, within the low-frequency section, a platform is present in the log(*σ*_ac_)–frequency curve. Fig. 14(b) gives the ratios of nanocomposite *σ*_ac_ to that of pristine epoxy resin (*σ*_ac Pri_) at 20 °C. It shows that nanocomposites own lower ac conductivities than pristine epoxy resin at low frequency. Fig. 14(c) shows the *σ*_dc_ values of all the composite samples under a dc electric field. Clearly, at a low temperature, *σ*_dc_ in the pristine epoxy resin is higher than in the nanofilled composites. Furthermore, *σ*_dc_ of the 3 wt% nanofilled composite is only 14% of that of the pristine sample at 80 °C. Nevertheless, if the sample temperature is >120 °C, *σ*_dc_ becomes the smallest in the pristine epoxy resin.

## 4. Discussion

The molecular weight of the CHBP used in this study is approximately 2600, which is greater than the molecular weight of most frequently-used silane coupling agents. Moreover, there are 12 terminal carboxyl groups. Because the carboxyl radicals can participate in the addition polymerization reaction,^25^ the nanosilica can not only combine with sidechains of the epoxy resin but also can be embedded in the main chains of the epoxy resin. This can significantly change the linking status of the nanofiller and epoxy resin molecules, modifying the dielectric behavior and relaxation properties.

As indicated by the TSDC experiments, the epoxy resin filled with CHBP-treated nanosilica releases a smaller TSDC and less charge than the unfilled epoxy resin after polarization under a dc electric field. Generally, two dominant reasons are identified for the appearance of the TSDC in insulating polymers such as epoxy resin. The first is the relaxation of the dipolar orientation. Because the epoxy resin matrix is composed of polar molecules, many dipoles are present, and relaxation of these dipoles can generate the TSDC. The second reason is the detrapping of space charges. During the manufacturing process of the composites, defects, interfaces, and impurity carriers should generate traps with different energies.^26^ The thermally assisted detrapping of trapped charges is inevitably introduced, and these charges act as carriers. The dipolar relaxation and the charge-detrapping process maintain a close relationship with the composite temperature and molecular bond strength.

The DSC test indicates that *T*_g_ of the epoxy resin composite increases remarkably by the addition of 3 to 7 wt% of the CHBP-treated nanofiller. Because the CHBP molecules react with epoxy resin radicals and increase the cross-linking degree, the diversity of epoxy resin anisotropy is reduced, and only one DSC peak is present. Additionally, trap-energy calculation suggests that the trap energy of the epoxy resin becomes obviously deeper by the CHBP-treated nanofiller. All of the aforementioned improvements are ascribed to the introduction of the bridge linkage of CHBP molecules, which enhanced the bond strength of the nanosilica and epoxy resin molecules. Deep traps help to inhibit the charge injection from electrodes and increase the energy needed for charge detrapping. The increased *T*_g_ also makes the relaxation or jumping of charges in epoxy resin molecules difficult.


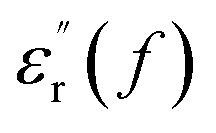
 curve of nanocomposites shows that obvious conduction process is present at high temperature. 
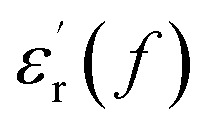
 curve also shows a giant permittivity at low frequency (<1 kHz) and an almost constant permittivity at high frequency (>1 kHz). Moreover, the real and imaginary permittivity curves approximately obey a log-linear frequency dependency at low frequency. Considering that the conduction process could not influence the real permittivity, the giant permittivity at very low frequency should be ascribed to low-frequency dispersion of nanocomposites. This means that at the low frequency range, the migration of charges limited in some local regions dominates the process of composite dielectric response.^27^ In 
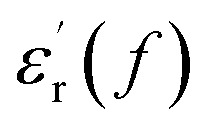
 curves, it also can be found that the initial dispersion frequency of epoxy resin composites is significantly decreased by introduction of nanosilica.

The experiment of ac conductivity shows that with addition of CHBP-treated nanosilica, *σ*_ac_ of nanocomposites is lower than that of pristine epoxy resin at low temperature (<120 °C). This may be deduced by deep traps and reinforced interface structure. As a result, the relative permittivity and dielectric loss of nanocomposite at low temperature are lower than those of pristine epoxy resin.

However, note that the slopes of 
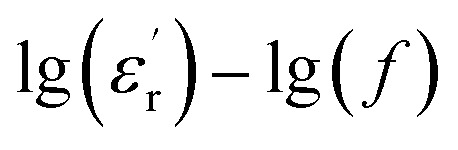
 and 
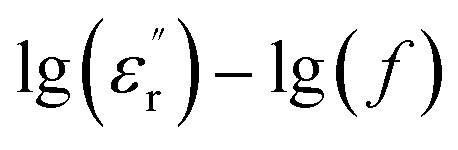
 curves are quite different, and clear relaxation peaks are present in the curve of 
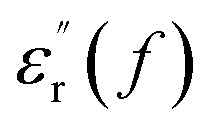
 and *M*′′(*f*). This suggests that the local electric conduction existing in composites is not the only one process which is responsible for nanocomposite dielectric response at low frequency.^28^ Owing to the existence of CHBP-treated nanosilica, interfacial polarization and dipole relaxation should be other two important processes which influence the dielectric response of nanocomposites.

Since the interfacial polarization of nanocomposites is sensitive to nanofiller content, when the filler content is kept below 5 wt%, the nanosilica is not close enough to the neighbouring filler for the nanofiller interface areas to overlap with each other, and the enhanced bond between the nanofiller and epoxy resin molecules can effectively inhibit the orientation of polar molecular chains or side groups in interfacial areas.^29^ However, if the filler content reaches a certain value, 7 wt% in this study, the adjacent overlapping of the nanofiller interface area occurs, reducing the bond strength of the nanofiller surface,^30^ and the regional interfacial polarization of high permittivity is enhanced.^31,32^ As a result, the composite relative permittivity is increased.

**Fig. 15:**
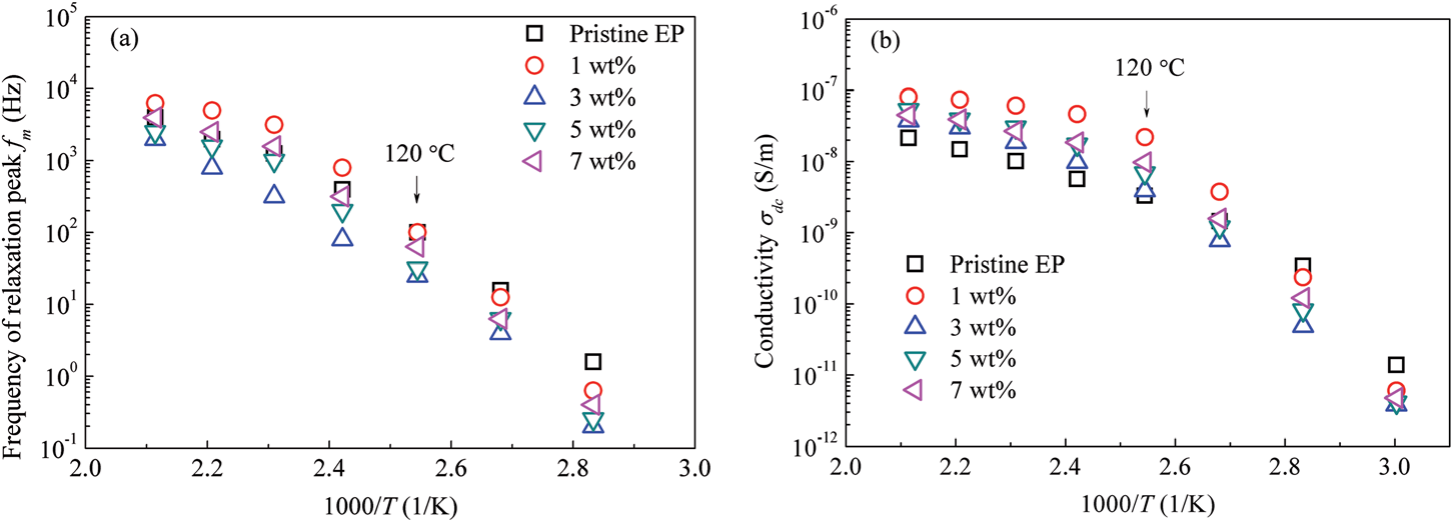
Peak frequency of the relaxation and dc conductivity as a function of the reciprocal temperature: (a) peak frequency of relaxation; (b) dc conductivity.

Moreover, the CHBP modifier changes the interfacial properties and influences the total relaxation mode of epoxy resin composites. Fig. 15 shows the peak frequency of the molecular relaxation (a) and dc conductivity (b) as a function of the reciprocal temperature. Clearly, log(*f*_m_)–1/*T* and log(*σ*_dc_)–1/*T* do not satisfy the Arrhenius relation at high temperature.

As it reported that the relaxation process of epoxy resin at high temperature can be described by the Vogel–Tammann–Fulcher (VTF) law,^33^ the relaxation of epoxy resin filled with CHBP-treated nanosilica may also satisfy the VTF law at high temperature. According to the VTF law, the temperature dependence of the peak frequency of relaxation and the dc conductivity can be expressed by eqn (2) and (3), respectively.^34^ Here, *f*_m0_, *A*_f_, *T*_f0_, *σ*_dc0_, *A*_dc_, and *T*_dc0_ are positive constants.2
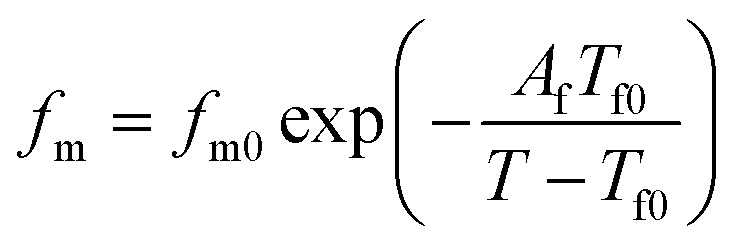
3
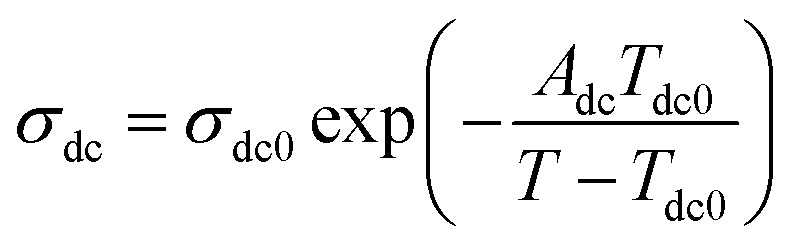


**Fig. 16:**
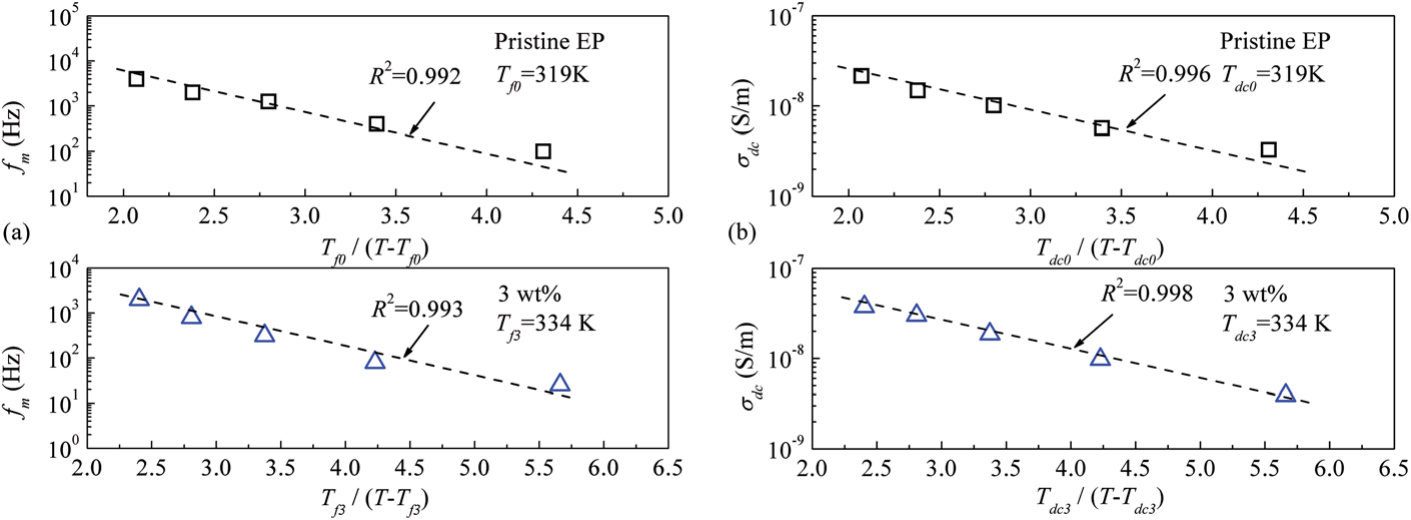
Peak frequency of the relaxation and dc conductivity as a function of the VTF-constructed temperature factor: (a) peak frequency of relaxation; (b) dc conductivity.

Fig. 16 shows the experimental data for the peak frequency of the relaxation (*f*_m_) and dc conductivity (*σ*_dc_) as a function of the constructed temperature factor described by eqn (2) and (3). The data indicate that fm and *σ*_dc_ are proportional to the exponential term, and the VTF law is valid in this case. This suggests that the molecular structure of the epoxy resin composites is thermally stretched to allow molecular chains to move at a high temperature, and the energies of the carriers are sufficient for transport in the composites. Because the *T*_f0_ or *T*_dc0_ calculated *via* VTF linear fitting is approximately 50 K lower than *T*_g_ of the composites,^35^ the VTF-fitted parameters are consistent with the DSC results.

Furthermore, the experiments in this study also indicate that if the operating temperature of the epoxy resin composite is higher than *T*_g_, the advantages of the low permittivity, low dielectric loss, and low dc conductivity due to the CHBP-treated nanosilica filler gradually disappear. This may be because of the composite structural change to the amorphous state, wherein it is easy for polymer molecular chains to move or turn. Thus, the structural equilibrium of the nanofiller interface is broken, and the CHBP polar molecules join in the interface polarization. Moreover, the bond strength of the nanofiller surface is reduced or undermined, thus, the detrapping and charge transport are enhanced. These thermal charges result in the increase in 
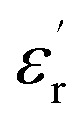
, 
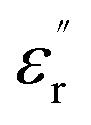
, *σ* in the composites at a high temperature.

## 5. Conclusions

Because of the terminal carboxyl radicals and the long molecular chain, CHBP is effective for the surface modification of nanosized inorganic fillers. In this study, the dielectric and relaxation properties of composites of epoxy resin and CHBP-treated nanosilica are investigated, and the following conclusions are drawn.

Processed by silane and hyperbranched polyester, nanosilica surface can be grafted with a functional polymer layer ranging in thickness from several to tens of nanometers. Owing to its big matrix molecular weight, the surface polyester layer can effectively inhibit the agglomeration of nanosilica particles in epoxy resin.

With addition of CHBP-treated nanosilica of 30 nm in diameter, deep traps with an energy of 1.09 eV are introduced in the epoxy resin composites. Real permittivity and dielectric loss of the nanocomposites of 10 mHz to 1 kHz is obviously decreased under low temperature, and the glass transition temperature of the composites is increased by 11 °C at most. Conductivity under ac and dc electric field are also lower than those of pristine epoxy resin, if the operating temperature is <*T*_g_.

The polarization process of epoxy resin nanocomposites filled with hyperbranched-polyester-treated nanosilica is dominated by regional carrier migration, interfacial and dipole polarization. Owing to the enhancing effect of hyperbranched polyester surface layer on bond strength of nanosized interfaces, the low-frequency dispersion deduced by regional charge movement and interfacial polarization in nanocomposites both are inhibited at low temperature. The relaxation frequency of dipole polarization in nanocomposites is also decreased, and the dipole relaxation procedure at high temperature (>*T*_g_) is transformed to satisfy the VTF relation.

## Conflicts of interest

There are no conflicts to declare.

## Supplementary Material
